# Physiological and therapeutic regulation of PCSK9 activity in cardiovascular disease

**DOI:** 10.1007/s00395-017-0619-0

**Published:** 2017-04-24

**Authors:** Simon Glerup, Rainer Schulz, Ulrich Laufs, Klaus-Dieter Schlüter

**Affiliations:** 1 Department of Biomedicine, Aarhus University, 8000 Aarhus C, Denmark; 2 Department of Physiology, Justus-Liebig-University, Aulweg 129, 35392 Giessen, Germany; 3 Universitätsklinikum des Saarlandes, 66421 Homburg/Saar, Germany

**Keywords:** LDL, oxLDL, LDL receptor

## Abstract

Ischemic heart disease is the main cause of death worldwide and is accelerated by increased levels of low-density lipoprotein cholesterol (LDL-C). Proprotein convertase subtilisin/kexin type 9 (PCSK9) is a potent circulating regulator of LDL-C through its ability to induce degradation of the LDL receptor (LDLR) in the lysosome of hepatocytes. Only in the last few years, a number of breakthroughs in the understanding of PCSK9 biology have been reported illustrating how PCSK9 activity is tightly regulated at several levels by factors influencing its transcription, secretion, or by extracellular inactivation and clearance. Two humanized antibodies directed against the LDLR-binding site in PCSK9 received approval by the European and US authorities and additional PCSK9 directed therapeutics are climbing up the phases of clinical trials. The first outcome data of the PCSK9 inhibitor evolocumab reported a significant reduction in the composite endpoint (cardiovascular death, myocardial infarction, or stroke) and further outcome data are awaited. Meanwhile, it became evident that PCSK9 has (patho)physiological roles in several cardiovascular cells. In this review, we summarize and discuss the recent biological and clinical data on PCSK9, the regulation of PCSK9, its extra-hepatic activities focusing on cardiovascular cells, molecular concepts to target PCSK9, and finally briefly summarize the data of recent clinical studies.

## Introduction

Proprotein convertase subtilisin/kexin type 9 (PCSK9) regulates the low-density lipoprotein (LDL) receptor (LDLR) by a mechanism that appears to be rather unique in biology. However, despite the high current interest, important details remain to be understood about how PCSK9 mediates LDLR degradation.

### PCSK9 shifts LDLR traffic from recycling to degradation in hepatic cells

Proprotein convertase subtilisin/kexin type 9 is a member of the proprotein convertase superfamily of serine proteinases also encompassing proprotein convertases 1, 2, 4, 5, 7, furin, paired basic amino-acid cleaving enzyme 4, and subtilisin kexin isozyme-1 [176]. The only PCSK9 substrate identified so far is its own prodomain: ProPCSK9 undergoes autocleavage at position Gln^152^ and this is a prerequisite for subsequent passage through the secretory pathway [12]. Interestingly, the cleaved prodomain remains firmly attached by strong hydrophobic interactions in the putative substrate-binding cavity of the catalytic domain, thereby preventing PCSK9 from interacting with other substrates. Instead, PCSK9 binds to the extracellular domains of a highly selective subset of transmembrane receptors including LDLR and targets them for degradation in lysosomes by a mechanism that apparently is independent of PCSK9 proteolytic activity [170, 173]. The LDLR binds LDL cholesterol (LDL-C) and removes it from the circulation by endocytosis via clathrin coated pits. The acidic pH of endosomes causes LDLR to dissociate from LDL-C. LDLR recycles to the cell surface, while the LDL-C particle is degraded in lysosomes and the recovered cholesterol is used by the cell. LDLR bound to PCSK9 is also endocytosed by a clathrin-dependent mechanism, but the strength of the binding is increased at acidic pH, and instead of recycling, the entire complex is destined for lysosomal degradation [141, 203, 204]. Accordingly, overexpression of PCSK9 in experiments in mice, hamsters, and pigs results in reduced number of LDLR accompanied by marked increases in plasma LDL-C [5, 16, 118, 120, 141]. In contrast, PCSK9 knockout mice are characterized by increased LDLR expression and reduced LDL-C [148]. Similarly, genome editing, using a clustered regularly interspaced short palindromic repeats (CRISPR)/CRISPR-associated system, effectively down-regulates hepatic expression of PCSK9 with the subsequent expected up-regulation of LDLR and reduction in plasma LDL-C in mice [41].

### From genetics to function

A few months after the initial publication on PCSK9 by Seidah and colleagues [174], the first report on gain-of-function (GOF) mutations in PCSK9 causing autosomal dominant hypercholesterolemia was published [1]. This initial finding rapidly prompted several other genetic studies linking PCSK9 mutations with familial hypercholesterolemia [103, 180, 192] and the pivotal discovery in 2004 that PCSK9 overexpression in the liver resembles an LDLR knockout phenotype [120]. In 2005, loss-of-function (LOF) PCSK9 mutations including two nonsense mutations (Y142X and C679X) were discovered, conferring reduced LDL-C [31] and protecting against coronary artery disease [30]. PCSK9 GOF mutations with known functional consequences include the S127R and D374Y variants which display several folds increased affinity for LDLR [35]. Other PCSK9 GOF variants are R218S and F216L, and these are resistant to furin-mediated proteolytic inactivation as discussed below [13]. Furthermore, although the mechanism is currently unclear, a number of known GOF variants are associated with increased passage of PCSK9 through the biosynthetic pathway. Conversely, a number of LOF variants display reduced processing and exit from the endoplasmic reticulum as a consequence of compromised folding and stability [11, 133]. Studies of GOF and LOF PCSK9 mutations provided valuable insights into the biological activity of PCSK9 and lay the foundation for the development of therapeutic PCSK9 inhibitors.

### Contributions of N- and C-terminal domains to PCSK9 activity

PCSK9 consists of three domains: the N-terminal prodomain followed by the catalytic domain and the C-terminal domain. The catalytic domain mediates the direct interaction with the epidermal growth factor (EGF) A domain of the LDLR as demonstrated by various biochemical methods including crystallography [35, 94, 115]. However, elements of the PCSK9 prodomain appear to have a modulatory effect on LDLR degradation activity as deletion of the amino-acid sequence stretch 31–58 in the prodomain results in a PCSK9 variant with four to sevenfold increased LDLR degrading activity. The prodomain sequence is rich in acidic residues and has been suggested to play an autoinhibitory role through interaction with the basic residues in the PCSK9 catalytic domain [14]. Another possible regulatory function of the acid prodomain sequence has been proposed as around 40% of PCSK9 in plasma is associated with LDL-C particles and in vitro prodomain truncation variant lacking residues 31–52 is unable to bind LDL-C [207]. Furthermore, in cell-based experiments LDL-C particles lower the LDLR degradation activity of PCSK9 [91]. Thus, high LDL-C may provide a feedback mechanism to decrease PCSK9 activity. This effect is clearly not decisive as evident from the potent effect of PCSK9 inhibitors in lowering LDL-C in clinical trials (see section “Studies of PCSK9-inhibition in patients with high cardiovascular risk”).

The C-terminal domain of PCSK9 is required for its ability to induce LDLR degradation as a truncated PCSK9 variant lacking the C-terminal domain display reduced activity against LDLR, whereas the isolated C-terminal domain alone has no effect on LDLR degradation [210]. The role of the PCSK9 C-terminal domain is strongly supported by data using a monoclonal antibody directed against the C-terminal domain which inhibits the ability of PCSK9 to reduce LDL-C uptake in cells and decreases plasma LDL-C in cynomolgus monkeys [134, 165]. Notably, amyloid precursor-like protein 2 (APLP2) acts as a PCSK9 receptor as demonstrated by affinity purification from HepG2 cells followed by mass spectrometry. APLP2 binds to the PCSK9 C-terminal domain with an increasing amount of complex formed at decreasing pH. APLP2 mediates PCSK9 endocytosis alone or in complex with LDLR, and is critically involved in the lysosomal targeting of PCSK9 and the PCSK9:LDLR complex [39, 40]. Similarly, annexin-2 binds to the C-terminal domain of PCSK9 and functions as an endogenous inhibitor of its activity [120]. Accordingly, mice lacking annexin 2 displayed increased plasma PCSK9 and LDL-C and reduced LDLR, while overexpression of annexin 2 increased liver LDLR [176].

### The PCSK9 receptor X

Since the discovery of PCSK9-induced LDLR degradation, it has been a puzzle how a soluble monomeric protein like PCSK9 can have a dramatic effect on the intracellular trafficking of a transmembrane type 1 receptor such as LDLR. Furthermore, the LDLR C-terminus is not absolutely required for PCSK9-induced degradation. LDLR-transferrin receptor chimera with the LDLR C-terminal substituted for that of the transferrin receptor domain as well as a truncated LDLR variant lacking the C-terminus are still degraded albeit at slower rates [78, 186]. The average PCSK9 plasma concentration is around 6 nmol/L, but the affinity constant (*K*_d_) for the PCSK9:LDLR interaction at a pH corresponding to that of plasma has been measured using Biacore to be around 170 and 628 nmol/L, respectively [35, 53, 95]. Interestingly, the same studies found that at lower pH, the affinity was markedly increased and the reported *K*_d_ values were 62 nmol/L (pH 5.4) [35] and 4.2 nmol/L (pH 5.3) [53]. Taken together, these observations suggest the existence of additional high affinity PCSK9 receptors that take part in the LDLR degradation complex.

## Molecular pathways regulating PCSK9 expression

Proprotein convertase subtilisin/kexin type 9 expression is highly restricted both developmentally and in tissues [174]. In adult mice, the highest PCSK9 mRNA levels by far are found in the liver and substantially lower expression is also found in the brain, kidney, and small intestine [191]. Thus, circulating PCSK9 appears to be produced mainly by the liver and its expression is regulated by numerous factors such as diurnal rhythm, hormones (including estrogen, insulin, resistin, and thyroid hormone), diet, exercise, and various cholesterol lowering drugs.

### Regulation of PCSK9 expression by statins

Soon after the discovery of PCSK9, it was reported that statins up-regulate PCSK9 gene expression in HepG2 cells and primary hepatocytes [48]. PCSK9-deficient mice are hypersensitive to statin treatment compared to wild-type mice [148]. These observations from cells and animals have translated into humans: a first report of patients receiving atorvastatin showed a 34% increase in circulating PCSK9 levels in response to treatment [25] and a recent meta-analysis including 15 clinical trials examining the effect of statins on plasma PCSK9 levels showed that statins significantly increase plasma PCSK9 irrespective of the type of statin [164]. Interestingly, several additional lipid-lowering drugs including fenofibrates increase PCSK9 expression and thereby counteract their own pharmacological activity [163, 195].

### Regulatory elements in the PCSK9 promoter

**Fig. 1:**
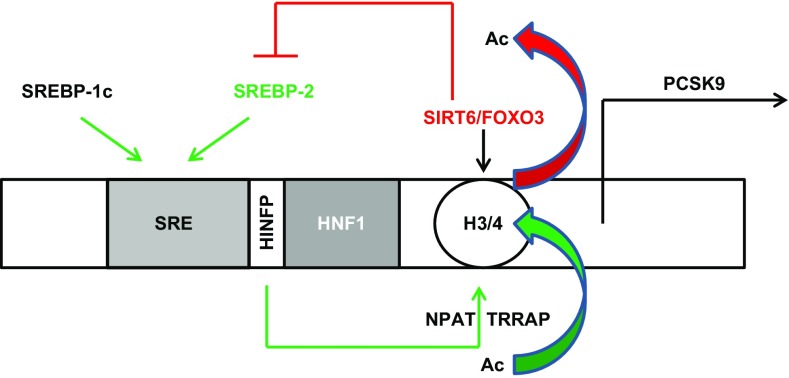
Regulation of PCSK9 promoter region in hepatic cells. Pathways that increase the expression are indicated in* green* and those that depress expression of PCSK9 are indicated in* red*. The sterol-regulatory element (SRE) is activated by SREBP1c and SREBP2 that coactive hepatocyte nuclear factor (HNF)-1. The histone H3 is deacetylated by SIRT6/FOXO3 complexes and histone H4 is acetylated by HINPF/NPAT/TRRAP complexes

Up-regulation of PCSK9 by cholesterol depletion or inhibition of intracellular cholesterol synthesis by, e.g., statins is explained by a sterol-regulatory element (SRE) [48], which have found to be regulated by sterol-regulatory element-binding protein-2 (SREBP-2) and SREBP-1c [32, 81, 157]. In close vicinity to the SRE, the PCSK9 gene contains a highly conserved hepatocyte nuclear factor 1 (HNF1)-binding site and HNF1α cooperates with SREBP-2 to regulate PCSK9 expression in HepG2 cells [107] and in the liver [45, 179]. Furthermore, liver-specific knockdown of HNF1 antagonizes statin-induced induction of PCSK9 transcription [44]. In analogy, mice with liver-specific knockout of SREBP-2 display a marked reduction in PCSK9 expression. A binding site for histone nuclear factor P (HINFP) is located between the SRE and HNF1 site in the PCSK9 promotor. HINFP was suggested as a positive regulator of PCSK9 transcription through facilitation of histone H4 acetylation at the PCSK9 promoter through a complex of HINFP, its cofactor nuclear protein of the ATM locus (NPAT), and the histone acetyltransferase cofactor transformation/transcription domain-associated protein (TRRAP) [108]. Along this line, deficiency of the histone deacetylase Sirtuin 6 (Sirt6) in the liver leads to elevated PCSK9 expression. As underlying mechanism recruitment of Sirt6 to the PCSK9 promoter by the transcription factor forkhead-box protein O3 (FoxO3), leading to deacetylation of histone H3, thereby repressing PCSK9 transcription by suppressing HNF1 activity [190]. Furthermore, Sirt6 and FoxO3 also suppress hepatic SREBP-2 transcription [189]. Interestingly, Sirt6 and FoxO3 are active during fasting but decrease upon feeding, and both genes are associated with longevity, possibly through their effect on LDL-C clearance [190]. In accordance with these findings, PCSK9 expression decreases by fasting by a mechanism that is apparently insulin-independent [32, 125]. Furthermore, the pharmacological activation of the related deacetylase Sirt1 leads to a marked reduction in PCSK9 secretion through an unknown posttranslational mechanism [126]. Figure 1 summarizes some aspects of regulatory elements of the PCSK9 promoter mentioned here.

### Insulin signaling, diabetes, and PCSK9 expression

Constitutive expression of PCSK9 and LDLR has been demonstrated in pancreatic islet β cells [122]. Here, depletion of PCSK9 also increases LDLR, but it is also associated with low insulin expression. Pancreatic islet β-cells in PCSK9 null mice show all signs of inflammation, apoptosis, and malformation. PCSK9 expression is associated with insulin signaling, possibly through an effect of insulin on SREBP-1c [32]. Insulin is reported to increase PCSK9 expression in hepatoma cells and primary hepatocytes [32, 125], while another study found that insulin decreases PCSK9 expression and secretion from human cell lines [8]. In humans, plasma PCSK9 concentrations are positively associated with insulin concentration [93] and insulin resistance [75, 104]. PCSK9 expression is markedly reduced in mice lacking the insulin receptor in the liver with pharmacologically induced insulin deficiency. However, insulin receptor knockdown in the liver was only effective in reducing PCSK9 expression in obese mice but not in lean wild-type mice [125]. Instead, in a different study, insulin repressed hepatic PCSK9 expression via activation of insulin receptors and subsequent activation of protein kinases finally leading to protein kinase C (PKC)δ-dependent inhibition of HNF4α and HNF1α [3]. Indeed, hepatic expression of PCSK9 is reduced under diabetic conditions, whereas hepatic LDLR expression remains high, but obviously functional inactive [136]. In vivo studies showed that hyperinsulinemic euglycemic clamp resulted in a marked increase in PCSK9 expression in mice [32], whereas a moderately hyperinsulinemic glucose clamp for 24 h had no effect on PCSK9 plasma concentrations in healthy and type 2 diabetic men [84]. In a different study, PCSK9 plasma levels dropped acutely following hyperinsulinemic euglycemic clamp in obese postmenopausal women confirming the mouse data [125], suggesting that PCSK9 levels are dynamically regulated following an insulin or glucose challenge [8]. Importantly, there is evidence for sex-specific regulation of PCSK9 in young adults [105]. Thus, it seems that the outcome of insulin signaling on PCSK9 expression is highly sensitive to the experimental setup and the exact metabolic state of the animals or human subjects used for testing. High fructose diet increases circulating PCSK9 both in healthy human subjects [26] and in animals with concomitant effect on LDLR protein levels [43]. Having summarized these experimental data, it has to be mention that PCSK9 variants associated with lower LDL-C displayed an increased type-2-diabetes risk [167]. Furthermore, in type-1-diabetes, good glycaemic control abolishes the relationship between PCSK9 and LDL-C [101] indicating a complex and yet not fully interaction between glucose-, LDL-C, and PCSK9 regulation. Nevertheless, there is no evidence yet for new onset of diabetes in patients under PSCK9 inhibition [6].

### Circadian regulation of PCSK9 expression

**Fig. 2:**
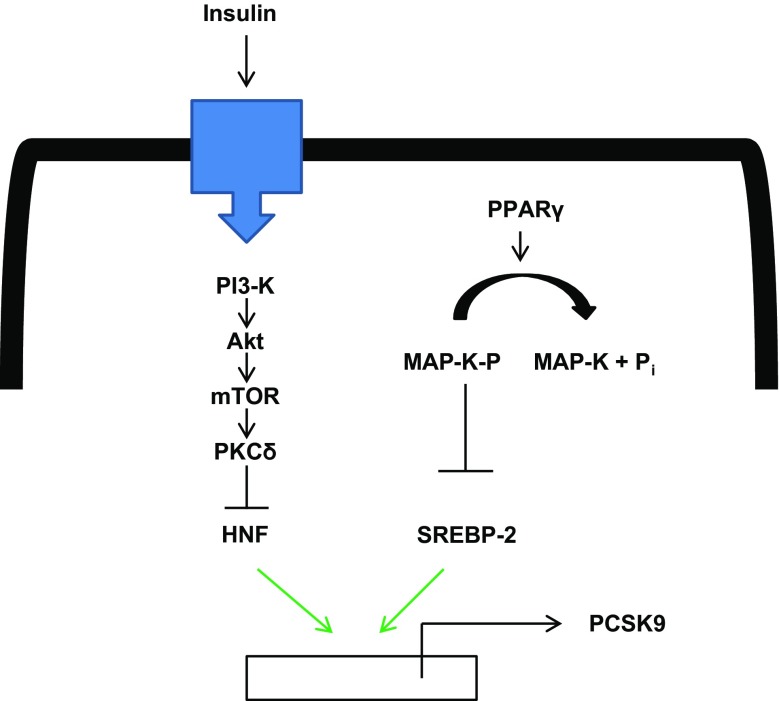
Effect of insulin and PPARγ on PCSK9 expression. Insulin activates a classical insulin receptor signaling cascade leading to a depression of HNF1 and thereby PCSK9 expression. PPARγ activates PCSK9 transcription by attenuating MAP-kinase-dependent brake on PCSK9 expression

Changes in plasma PCSK9 levels are synchronous with cholesterol synthesis under noninterventional conditions. Interestingly, disruption of the circadian rhythmicity of liver gene expression in liver-specific aryl hydrocarbon receptor nuclear translocator-like protein 1 (=Bmal1) knockout mice results in marked increases in PCSK9 levels and concomitantly reduced LDLR with subsequent increase of LDL-C [117]. Bmal1 targets the transcriptional regulation of hundreds of genes including all of the clock genes and a number of genes encoding metabolic regulators. Loss of Bmal1 markedly reduces expression of the Tribbles homolog 1, a protein involved in the turnover of transcription factor CCAAT/enhancer-binding protein α (C/EBPα) and also in the phosphorylation and activation of mitogen activated protein (MAP) kinase both resulting in the inhibition of lipogenic gene transcription [10], including PCSK9 [117]. Interestingly, variants near the TRIB1 gene locus have repeatedly been genome-wide significantly associated with all plasma lipid traits [64]. Peroxisome proliferator-activated receptor γ (PPARγ) activation induces PCSK9 transcription. MAP kinase activation decreases PCSK9 expression, which is possibly through PPARγ dephosphorylation. Accordingly, PCSK9 expression in HepG2 cells is induced by MAP kinase inhibition [47]. The effect of PPARγ activity on PCSK9 transcription is associated with increased processing of SREBP-2 [47]. The effects of insulin and PPARγ on the regulation of PCSK9 expression are summarized in Fig. 2.

### Cross talk between cytokine signaling and PCSK9 expression

Interestingly, cytokines such as tumor necrosis factor (TNF)α or activation of the Janus kinase/signal transducer and activator of transcription (JAK/STAT) pathway has notable effect on PCSK9 expression in HepG2 cells and in vivo. As such, pharmacological activation of this pathway suppresses PCSK9 transcription [24]. On the other hand, suppressor of cytokine signaling SOCS3, which is a negative regulator of JAK/STAT, induces PCSK9 expression through activation of SREBP-1 [159]. Furthermore, the inflammatory cytokine resistin induces PCSK9 expression in human hepatocytes [124]. Intriguingly, the PCSK9 C-terminal domain displays structural resemblance to resistin suggesting both a structural and functional relationship between cytokine signaling and PCSK9 [73]. These observations are interesting considering that PCSK9 recently was described as a critical regulator of the innate immune response and survival following sepsis in both mice and humans [200].

In conclusion, several factors regulate PCSK9 expression, but it remains to be understood what controls the tissue specificity and the developmentally restricted PCSK9 expression.

## Posttranslational regulation of PCSK9 activity

Recently, it has become clear that PCSK9 activity is tightly regulated at the posttranslational level by a number of factors that regulates its secretion, extracellular inhibition or inactivation, and its clearance from the circulation.

### PCSK9 regulatory check points in the secretory pathway

In addition to the extracellular pathways, PCSK9 may also function intracellularly by sequestering LDLR in its biosynthetic pathway as PCSK9 and LDLR are both synthesized by hepatocytes. Of note many of the naturally occurring LOF mutations in PCSK9 result in altered proPCSK9 processing or reduced secretion from the cell, whereas mutations associated with hypercholesterolemia resulted in increased cellular exit of PCSK9 [133]. The mechanism for this remains unknown but suggests that the passage of PCSK9 through the biosynthetic pathway is tightly controlled. Understanding of the pathway may be of clinical importance to assess potential differential effects of extracellular PCSK9 inhibitors such as antibodies and inhibitors with intracellular effects.

### PCSK9 in the endoplasmic reticulum

The chaperone heat-shock protein 90 kDa β member 1 (=GRP94) residing in the endoplasmic reticulum (ER) binds PCSK9 and prevents its ability to sequester LDLR prematurely in the ER [145]. Mice lacking GRP94 have increased plasma LDL-C and a highly reduced LDLR level in the liver. Proteins destined for secretion can either exit the ER by the so-called bulk flow pathway or they can be guided by specific ER-residing sorting receptors [36]. The ER sorting receptor for PCSK9 is the coat protein complex II (COPII) vesicle adaptor protein SEC24A, since it is required for efficient exit of PCSK9 from the ER [29]. SEC24A is highly enriched in the liver and SEC24A knockout mice display reduced plasma PCSK9 and LDL-C but increased liver LDLR. COPII vesicles cycle between ER and the cis-Golgi and receptors are known to direct specific cargo into these vesicles, thereby increasing the speed of their ER exit. This traffic is again guided by specific adaptor proteins such as SEC24A. However, in the case of PCSK9 and SEC24A, the receptor remains to be identified. Endoplasmatic stress on the other hand reduces PCSK9 secretion [102].

### Sorting of PCSK9 by sortilin

Sortilin 1 (SORT1) encoding the transmembrane type I receptor sortilin is a risk gene for hypercholesterolemia and myocardial infarction [7, 87, 90]. Sortilin is highly expressed in a number of tissues in a distinct and dynamic fashion [17]. It is involved in cellular processes including cell death and survival as well as regulation of neurotrophic activity in the peripheral and central nervous system [66]. Sortilin in the liver, macrophages, and smooth muscle cells promotes atherosclerotic plaque formation in mice by apparently independent mechanisms [65, 72, 89, 130]. Sortilin was recently identified as a high affinity receptor for PCSK9 [72]. The affinity constant is in the low nanomolar range and close to the actual plasma concentration of PCSK9 [95]. Thus, the sortilin:PCSK9 interaction is up to 100-fold stronger than the interaction between LDLR and PCSK9 [35, 53]. Sortilin consists of a large extracellular domain followed by a single pass transmembrane region and a short cytoplasmic tail harboring sequence motifs for interactions with cytosolic adaptor proteins [66]. Unlike LDLR, sortilin is not destined for lysosomal degradation by PCSK9. Instead, sortilin appears to be involved in intracellular trafficking of PCSK9 as sortilin knockout mice display reduced level of circulating PCSK9, reduced LDL-C, and increased liver LDLR [72, 89]. On the other hand, viral overexpression of human sortilin in the mouse liver results in increased circulating PCSK9, increased LDL-C, and reduced liver LDLR. Interestingly, PCSK9 binding to sortilin is inhibited by the sortilin propeptide [72]. Since pro-sortilin is converted into its mature form by furin-mediated proteolytic processing in the trans-Golgi network (TGN) [132], it seems unlikely that sortilin and PCSK9 interact in the ER or cis-Golgi. The TGN is the main sorting hub of the cell and proteins may here be destined for constitutive or regulatory secretory vesicles, or to the different endosomal structures [37]. Hence, it is tempting to speculate that sortilin may play a decisive role in the TGN in directing PCSK9 for the secretory pathway in an efficient manner. A recent study reported that sortilin is a target for PCSK9-induced degradation but employed a sortilin variant with a C-terminal myc-tag [20]. The presence of any C-terminal tag in sortilin is known to affect its interaction with cytosolic adaptor proteins and may thus have a relevant effect on its cellular function and fate [33].

Interestingly, PCSK9 serum concentrations correlate positively with the sortilin extracellular domain (ECD) [72] which is released from the cell surface by a disintegrin and metalloproteinase (ADAM) both in a regulated and constitutive manner [50, 77]. Whether the sortilin ECD in plasma reflects increased expression levels of sortilin and thereby increased PCSK9 secretion or whether sortilin ECD has a PCSK9 modulatory activity on its own is currently unknown [79]. Interestingly, sortilin ECD acts as potential decoy receptor for the neurotrophic factor progranulin [146]. The expression of sortilin is modulated by a number of factors including diet and exercise, but notably, plasma sortilin ECD correlates with increased body mass index [21].

Taken together, sortilin is a high affinity receptor for PCSK9 that regulates PCSK9 at several levels by facilitating its trafficking in the TGN and possibly as a shedded form in plasma where it may form a complex with PCSK9. Furthermore, sortilin might affect PCSK9 indirectly by affecting cytokine signaling. However, the direction of the regulation is complex as sortilin influences cytokine signaling by several mechanisms and the sortilin effect on PCSK9 may, therefore, dependent on the tissue and the cellular context. Sortilin forms a physical complex with leukemia inhibitory factor (LIF) receptor-β and its presence enhances STAT3 phosphorylation induced by helical type I cytokines [97], a pathway that is reported to inhibit PCSK9 expression [24, 159]. Furthermore, sortilin directly affects both endocytosis as well as the secretion of several cytokines and inflammatory mediators from T-cells and macrophages [76, 97, 130].

### Extracellular regulation of PCSK9 by proteolytic inactivation

Mature PCSK9 can be detected in plasma and hepatocyte culture supernatant as a 62 kDa active form and a 55 kDa form with reduced activity towards LDLR. The lower molecular weight form originates from proteolytic processing at Arg^218^ by the related proprotein convertase furin and to a lesser extent by protein convertase 5 or 6A [13, 74]. This is highly interesting as the naturally occurring GOF PCSK9 variants R218S and F216L display altered proteolytic processing [13]. In the R218S variant with arginine at position 218 substituted for serine, the cleavage site is obstructed and no furin-mediated inactivation is observed. In the case of F216L, proteolytic inactivation is reduced, possibly because the sequence adjacent to the actual cleavage site no longer fits in the substrate-binding pocket of furin. Accordingly, plasma from homozygous individuals carrying R218S or F216L, respectively, has markedly reduced levels of the 55 kDa form of PCSK9 [49]. From a pharmacological point of view, it is also interesting that fibrates up-regulate furin and possibly increasing proteolytic PCSK9 inactivation [92].

Furin cleavage likely primes PCSK9 for further degradation and thereby elimination from the circulation. Recently, matrix metalloproteinase 2 was also found to proteolytically inactivate PCSK9. However, the physiological relevance of this regulatory effect remains to be established [202].

### Clearance mechanisms of plasma PCSK9

LDL receptor plays a major role in clearance of plasma PCSK9. In wild-type mice, iodinated PCSK9 has a half-life of approximately 5 min, while this is increased to 15 min in LDLR knockout mice [67]. The radioactivity mainly accumulates in the liver, but a significant portion is found in the kidney [191]. However, PCSK9 is still relatively rapidly removed from the circulation in the absence of LDLR, suggesting the existence of other clearance mechanisms. This is supported by the fact that individuals homozygous for LDLR inactivating mutations have markedly increased LDL-C, but the PCSK9 levels are not correspondingly high [23]. One obvious clearance mechanism is proteolytic degradation of PCSK9 initiated by furin as described above. Another proposed PCSK9 clearance receptor is amyloid β precursor like protein 2, but this remains to be established in vivo [39, 40].

## PCSK9 effects distinct from hepatic LDLR regulation

Without any doubt, the main overall physiological effect of PCSK9 is that on hepatic expression of LDLR thereby controlling the plasma concentration of LDL-C and indirectly also that of oxidized LDL (oxLDL) [138]. However, hepatic regulation of LDLR by PCSK9 has consequences on other target tissues that respond to LDL-C and oxLDL. Furthermore, PCSK9 is also expressed in extra-hepatic tissues such as heart and kidney. In most extra-hepatic tissues, cellular cholesterol concentration is less dependent on LDLR-driven cholesterol uptake, because most peripheral tissues regulate their cholesterol ester levels by secretion rather than uptake of LDL-C [138]. Nevertheless, extra-hepatic cells express LDLR that might be regulated by PCSK9, too. In extra-hepatic cells, LDL-C and oxLDL are agonists for mainly three different receptors: LDLR, low-density lipoprotein receptor-related protein 1 (LRP-1 = CD91), and lectin-like oxidized LDL receptor 1 (LOX-1). Their endogenous expression is controlled by PCSK9 and the concentrations of their ligands are controlled by PCSK9 as well. Finally, PCSK9 can exert effects that are independent from the surface expression of LDLR as it also interferes with intracellular molecule transport of very low-density lipoprotein (VLDL) receptors, cluster of differentiation 81 (CD81), and epithelial sodium channel (ENaC) [170]. Furthermore, in the intestine, for example, GOF mutations of PCSK9 up-regulate cholesterol transporters NPC1L1 and CD36, thereby increasing cholesterol uptake in an LDLR independent way [106]. In this section, examples for extra-hepatic effects of PCSK9 are briefly summarized and those most relevant for cardiovascular aspects are highlighted. The role of PCSK9 in glycaemic control and diabetes will not be discussed, because the most important findings are already summarized in section “Insulin signaling, diabetes and PCSK9 expression”.

### PCSK9 and its role in lipopolysaccharide (LPS)-driven inflammation

Inflammatory processes are involved in a variety of known cardiovascular diseases such as infection, hypertension, atherosclerosis development, and ischemia/reperfusion injury. LRP-1 antagonizes the pro-inflammatory effects of classical triggers of inflammation like LPS and TNFα. While activation of plasma membrane-bound LRP-1 depresses nuclear factor kappa-light-chain-enhancer of activated B cells (NFκB) signaling, extracellular LRP-1 activates stress activated kinases like p38 MAP kinase [119]. While the activation of LRP-1 attenuates LPS-driven inflammation, LPS itself increases the expression of PCSK9 and, therefore, decreases the hepatic expression of LDLR [51, 68]. This leads to increased plasma LDL-C and indirectly oxLDL, as well. oxLDL itself induces a pro-inflammatory process in macrophages depending on the activation of NFκB pathways. oxLDL increases the expression of PCSK9 in macrophages and the effects of oxLDL on NFκB activation depend on the induction of PCSK9 [188]. On the other hand, PCSK9 inhibits expression and activity of Abca1 protein in macrophages, thereby reducing cholesterol efflux of the cell [2]. Collectively, there are different levels at which PCSK9 interferes with the consequences of inflammation and modifies the overall response. For a review about the relationship between inflammation and PCSK9, see Ref. [18].

### PCSK9 in vascular cells

Smooth muscle cells and to a lower extent also endothelial cells constitutively express PCSK9 [42, 52]. The functional relevance is not clear. The vascular expression of PCSK9 is considerable high in regions with low shear stress, i.e., aortic branching regions and may correlate with higher levels of reactive oxygen species (ROS) and inflammation. In patients with acute coronary syndrome, high plasma levels of PCSK9 correlate with inflammation [61]. PCSK9 exerts direct effects on plaque composition [212], specifically on local inflammation and this is independent from LDL-C but related to LDLR expression [63]. Furthermore, platelet count is positively associated with plasma PCSK9 in coronary artery disease (CAD) patients, but the association is lost after adjustment for inflammation, again supporting a role for PCSK9 in inflammation [109].

Direct effects of oxLDL on endothelial cells were analyzed in the context of endogenous regulation of PCSK9 expression. oxLDL-dependent apoptosis was attenuated by siRNA directed against PCSK9 [208]. Furthermore, oxLDL induced the expression of PCSK9 in these cells [208]. Collectively, the data suggest that even in endothelial cells, a cell type that obviously expresses very low levels of PCSK9 and PCSK9 may be causally involved in cellular effects induced by oxLDL.

In summary, PCSK9 may affect atherosclerosis via regulation of LDL-C and oxLDL plasma concentrations. These effects are extensively reviewed, i.e., by Pirillo et al. [144]. Some recent clinical studies have found a correlation between acute coronary syndrome and major adverse cardiovascular events suggesting a clinical relevant relationship between the pro-inflammatory activity of PCSK9 described above and clinical relevant readouts [61, 109].

### PCSK9 and its role in cardiac physiology and pathophysiology

There are currently no studies available that clarify a potential role for PCSK9 for the function and adaptation of cardiomyocytes. As already mentioned, PCSK9 indirectly affects cardiomyocytes by modulating the plasma concentrations of LDL-C and oxLDL. As effects of LDL-C and oxLDL on cardiomyocytes are less well reviewed as those to vascular cells, they will be briefly summarized below.

Only few experiments were performed to analyze effects of LDL-C on cardiomyocytes. Cardiomyocytes control their cholesterol homeostasis mainly by regulated cholesterol efflux [150]. However, they express LDLR, but LDLR in cardiomyocytes does not serve mainly as a cholesterol uptake molecule [113, 150]. Depleting cholesterol concentration in cardiomyocytes by administration of probucol, a cholesterol synthesis inhibitor, interrupts cholesterol/caveolin-1 interaction in the plasma membrane, leading to accelerated degradation of human ether related gogo (hERG) channels, thereby prolonging action potential duration. This effect can be normalized by LDL-C suggesting cholesterol uptake in cardiomyocytes [69]. Adult rabbit cardiomyocytes exposed to LDL-C show slightly increased calcium transients. Mechanistically, this requires transsarcolemmal calcium transport pathways [113]. These experiments suggest that LDL-C may improve cardiac function. On the other hand, at least in atrial-like cardiomyocytes (HL-1 cells, an immortalized cell line with some cardiac-specific differentiation), LDL-C decreases the expression of sarcoplasmatic reticulum (SR) calcium ATPase (SERCA) 2a, ryanodine receptor 2 (RyR2), and connexin-40 within 24 h. This effect of LDL-C reduced calcium transients and slowed conduction velocity in such cells [9]. It remains elusive whether or not this difference is due to time differences between the studies (minutes vs. hours) or due to the source of myocytes (atrial vs. ventricular). Furthermore, the participation of PCSK9 in these effects of LDL-C has not been investigated.

Upon ischemia/reperfusion, cardiomyocytes accumulate cholesterol. In ischemic cardiomyopathy, activation of HIF-1α leads to up-regulation of LRP-1 [22, 27]. These receptors, unlike LDLR, seem to be responsible for cholesterol accumulation in cardiomyocytes. Additional cholesterol uptake seems to be achieved by VLDL receptors [142]. LRP-1 is not specific for LDL-C. α-Macroglobulin is another ligand for LRP-1 receptors in cardiomyocytes [139]. From these studies, it is already known that LRP-1 activation causes an activation of p42/p44 MAP-kinase pathways. Therefore, it must be expected that LDL activates p42/p44 MAP kinase in cardiomyocytes via interaction with LRP-1. LDL-C accelerates proteasomal degradation of dual specific phosphatases DUSP1 and DUSP6 in cardiomyocytes and thereby increases the amount of phosphorylated p42/p44 MAP kinase [60]. In addition, LRP-1 may directly activate p42/p44 MAP kinase pathways. LRP-1 receptors have an intracellular PKCα-binding domain [110] and PKCα pathways transiently activate p42/p44 MAP kinase in adult rat ventricular cardiomyocytes [169]. LRP-1 transiently moves to lipid drafts, cholesterol-enriched plasma microdomains that facilitate receptor signaling [158]. The data suggest that LDL-C via activation of LRP-1 receptors initially activates p42/p44 MAP kinase and that this activation is stabilized by induction of proteasomal-dependent degradation of DUSP1 and DUSP6. Of note, p42/p44 MAP kinase activation represses PCSK9 expression in hepatic cells. Extrapolating these data onto cardiomyocytes, activation of LRP-1 by LDL-C may potentially suppress PCSK9 expression.

Within the LRP receptor family, LRP-5 is activated during ischemia in hypercholesteremic animals and increased in its expression. Subsequent activation of the canonical Wnt-signaling pathway may protect against postischemic damage, because LRP-5 knockout mice had larger infarcts compared to wild-type mice [19]. Whether PCSK9 is involved in the myocardial effects of LRP-5 remains to be established.

Effects of oxLDL on cardiomyocytes are highlighted by findings that plasma oxLDL concentrations are associated with decreased cardiac function independent of vascular alterations [153]. Moreover, high oxLDL and high plasma levels of brain natriuretic peptide (BNP) are independent predictors of cardiac mortality [196]. Experiments on HL-1 cells showed that oxLDL induces the expression of BNP and also that of monocyte chemotactic protein (MCP)-1 in vitro [27]. MCP-1 is a well-established downstream target of NFκB, a signal transduction pathway that is involved in pro-inflammatory processes. Of note, LDL-C did not increase the expression of either BNP or MCP-1 in HL-1 cells. Therefore, effects are specific for oxLDL in myocytes and most likely evoked by activation of LOX-1. In addition, exposure of oxLDL to guinea pig cardiomyocytes caused severe electrophysiological and contractile dysfunction in these cells [213]. Again, these effects were not mimicked by LDL-C. Similar to LDL-C, low concentrations of oxLDL increase calcium transients most likely by activating translemmal calcium flux [114].

**Fig. 3:**
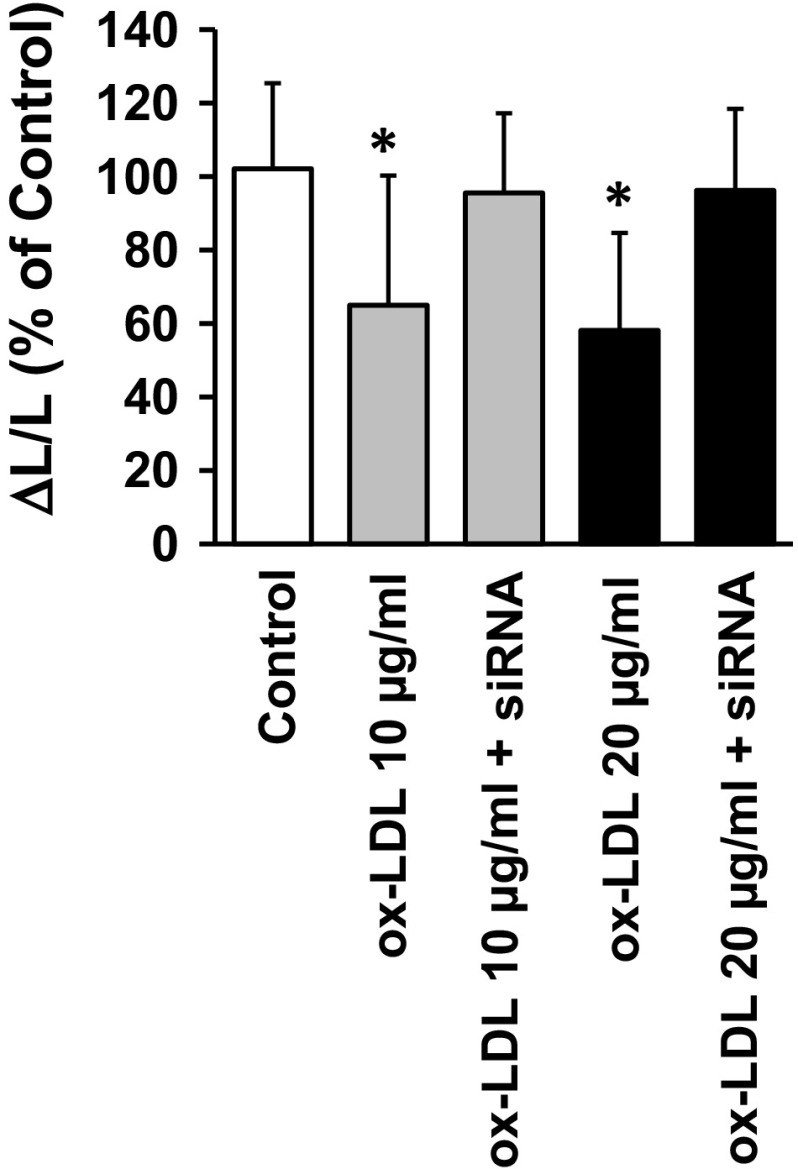
Effect of siRNA directed against PCSK9 on load-free cells shortening (Δ*L*/*L*) of isolated adult rat ventricular cardiomyocytes exposed to oxLDL. Data are mean ± SD from *n* = 90 cells (10 culture dishes; 2 preparations)

In cardiomyocytes oxidative stress, agonists of the renin-angiotensin-system (RAS), and oxLDL cooperate together. Angiotensin II induces the expression of BNP, causes cellular hypertrophy, and induces the expression of LOX-1. Activation of NFκB seems to trigger these events and angiotensin effects depend on LOX-1 expression [82, 83]. A coupling between LOX-1, angiotensin (AT) receptor 1 (AT_1_) expression, and AT_1_ activation suggests that LOX-1 is also involved in the onset of cardiac fibrosis [116]. Similarly, increasing oxidative stress to H9c2-cells by doxorubicin increases the expression of LOX-1 in cardiomyocyte-like cells. Activation of LOX-1 receptors subsequently induced cellular apoptosis [182]. oxLDL increases further the expression of calcium-sensing receptors (CaSR) that link extracellular calcium to intracellular calcium transients and contractility in cardiomyocytes [70, 168]. Whether the induction of apoptosis induced by doxorubicin requires an up-regulation and/or activation of CaSR is not known. However, once oxLDL has up-regulated CaSR, the subsequent activation of CaSR may lead to apoptosis. Reperfusion is another pathophysiological scenario leading to an increased expression of LOX-1 in cardiomyocytes. Neutralizing LOX-1 activation during reperfusion by administration of an anti-LOX-1 antibody reduces the infarct size suggesting that reperfusion does not only increase the expression of LOX-1 but also activates these receptors [86]. Collectively, several stress scenarios such as RAS activation, ischemia/reperfusion, and oxidative stress collectively increase the expression of LOX-1 in cardiomyocytes. Again, it remains to be established whether some of these oxLDL-dependent effects require PCSK9. Our unpublished data show that isolated adult rat ventricular cardiomyocytes constitutively express PCSK9 and that oxLDL-dependent effects on cell shortening can be antagonized by neutralizing PCSK9 in cardiomyocytes (Fig. 3). It is tempting to speculate that the aforementioned long-term effects of LDL-C on HL-1 cells on SERCA and RyR expression contribute to these effects. Moreover, it is unlikely that oxLDL directly damages cardiomyocytes [166]. However, oxLDL activates p38 MAP kinase via activation of LOX-1 and this attenuates cell shortening via down-regulation of SERCA [131].

### Conclusive remarks about the role of extra-hepatic effects of PCSK9 targeting

In summary, these emerging PCSK9 effects distinct from hepatic LDLR regulation described above need to be replicated and confirmed in humans. In addition, their importance for clinical outcomes will remain a matter of debate for some time for two main reasons: PCSK9-inhibiting antibodies lower LDL-C very potently which is likely the main effect in clinical trials and any extra-hepatic effects have to be established on top of LDL-C lowering. Second, regulatory agencies request that trials with PCSK9 inhibitors (PCSK9i) are performed on optimal statin therapy except for statin-intolerant patients. However, statins may mask some of the proposed effects. Here, comparing low-dose PCSK9i with high-potency statin matched in dose for equal LDL-C lowering may clarify the impact of PCSK9 independent of LDL-C-lowering effects.

## Approaches to reduce PCSK9 synthesis and/or PCSK9/LDLR interaction

PCSK9 interacts with LDLR intra-end extracellular domains and directs LDLR towards degradation. Therefore, to preserve LDLR expression, either the synthesis of PCSK9 or its interaction with the LDLR needs to be reduced. The interaction of PCSK9 with the LDLR can be attenuated by removing PCSK9 from the circulation (extracellular IgG antibody, monobodies, and vaccination) or offering alternative binding partners rather than LDLR (mimetic peptides) (for review, see Ref. [15]). The synthesis of PCSK9 can be reduced at the level of translation (silencing RNA and oligonucleotides) or its intracellular self-cleavage (mimetic peptides).

A large number of companies are currently involved in PCSK9 inhibitor research at preclinical and clinical trial stages (see https://citeline.com/pcsk9-inhibitors-future-hold-controversial-new-class/).

### Approaches targeting extracellular PCSK9/LDLR interaction

The most advanced approach for reducing LDLR expression is the administration of human monoclonal IgG antibodies and the recent clinical data are discussed later in this review article (see section “Studies of PCSK9-inhibition in patients with high cardiovascular risk”). The effects of PCSK9 antibodies are independent from the circulating PCSK9 concentration [38]. Apart from the therapeutic use of IgG antibodies, smaller molecular scaffolds targeting circulating PCSK9 are in development. This includes the development of single domain antibodies and antibodies directed against the catalytic domain of PCSK9 [178, 206].

Adnectins or monobodies are derived from the tenth extracellular type III domain of human fibronectin [112] and have been modified to target PCSK9 without affecting their structural stability [127]. In hypercholesterolemic, overexpressing human PCSK9 transgenic mice, a single intravenous injection of BMS-962476 (Bristol–Myers Squibb/Adnexus) reduced LDL-C and free PCSK9 levels. Treatment of cynomolgus monkeys with BMS-962476 suppressed free PCSK9 >99% within 10 min, resulting in reductions in LDL-C by approximately 55% within 48 h that persisted for nearly 3 weeks [127].

*Vaccines* Given the half-life of antibodies regular injections every 2–4 weeks are required. Therefore, peptide [59] or virus-like particle-based vaccines [34] targeting PCSK9 have been developed that stimulate the immune system to generate high-affinity, long-lasting PCSK9-specific antibodies. In mice [59] and macaques [34], a long-lasting decrease in circulating LDL-C up to 1 year was measured after application of the vaccines. Pharmaceutical companies (among them are Pfizer and AFFiRiS) developing PCSK9 vaccines already started (AFFiRiS) or will start clinical trials.

*Mimetic peptides* towards the PCSK9 binding motif on the LDLR (EGF-A) [123, 177, 211], the catalytic domain of PCSK9 [4], the prodomain of PCSK9 [140], and the C-terminal domain of PCSK9 [46] have been developed. A synthetic EGF-A mimetic peptide dose-dependently inhibited PCSK9-induced degradation of LDLR in HepG2 cells [177]. Also binding of PCSK9 to VLDL receptors was effectively inhibited by the synthetic EGF-A peptide [177]. Similarly, LDLR subfragments with a GOF mutation in the EGF-A binding motif (H306Y) blocked the binding of secreted PCSK9 to cell surface LDLR, thereby increasing LDLR expression in HepG2 cells [123]. Other mimetic peptides of 15-amino-acid length directed towards the disulphide loop (Cys323-Cys358) of PCSK9 containing the key GOF mutation D(374)/Y site exhibited a high LDLR promoting activity in both HepG2 and HuH cells [4]. As posttranslational modifications of PCSK9 increase its activity, mimetic peptides directed against the epitope phoshpho-Ser47 and sulpho-Y38 of PCSK9 also preserved LDLR levels in HepG2 cells [140]. Screening of phage-displayed peptide libraries identified a 13-amino-acid linear peptide (Pep2-8) as the smallest PCSK9 inhibitor. Pep2-8 bound to PCSK9 and fully restored LDLR surface levels and LDL particle uptake in PCSK9-treated HepG2 cells [209].

The development of fusion proteins that interact with the prosegment or the catalytic domain of the PCSK9/prosegment complex has been proposed for interference with PCSK9 processing and maturation. A recombinant fusion protein derived from the Fc portion of human IgG and containing the prosegment of PCSK9 directly bound to human PCSK9 [160]. Coincubation of HepG2 cells with the fusion protein and extracellular PCSK9 significantly attenuated PCSK9-mediated LDLR degradation, providing evidence that the fusion protein interferes with the effect of PCSK9 on LDLR at the extracellular level [160]. In addition, an imidazole-based compound was proven to inhibit PCSK9-LDL-R interaction thereby mediating a hypocholesterolemic effect [187].

PCSK9 binds to annexin A2 which prevents PCSK9-directed LDLR degradation in HuH7, HepG2, and Chinese hamster ovary cells [120]. Plasma analyses of annexin A2 knockout mice revealed an approximately 100% increase in PCSK9 levels and a 40% increase in LDL cholesterol levels, while adenoviral overexpression of annexin A2 in mouse liver increased LDLR expression in vivo [175]. Structure–function analyses demonstrated that the C-terminal cysteine–histidine-rich domain of PCSK9 interacts specifically with the N-terminal repeat R1 of annexin A2. Mutational analysis of this 70-amino-acid-long repeat indicated that the RRTKK81 sequence of annexin A2 is implicated in this binding, because its mutation to AATAA81 prevents its interaction with PCSK9 [120]. Thus, application of small mimetic peptides related to annexin A2 has also been proposed as a potential approach for PCSK9 inhibition [175].

Finally, a small peptide that impedes normal PCSK9 folding (SX-PCK9, Serometrix), thus hindering its binding to LDL receptors, is currently being studied.

Taken together—Small peptides directed against variable parts of PCSK9 reduce its interaction with the LDLR, thereby reducing LDL-C. Small peptides may offer the advantage of being orally applicable. However, there are no clinical trials testing the use of small peptides to inhibit PCSK9 at this time.

Interestingly, in this context, a cholesteryl ester transfer protein (CETP) inhibitor (K-312, Kowa) developed to primarily enhance HDL cholesterol also significantly reduced LDL-C in rabbits [128]. The mechanism identified in LDL-C reduction was a significant down-regulation of PCSK9. In addition, some natural occurring compounds might inhibit PCSK9 expression, such as lupin peptides or polydatin [96, 205].

### Genom editing and oligonucleotide-based therapeutics targeting intra- and extracellular PCSK9

Using a CRISPR-associated (Cas) 9 genome-editing system to target the human PCSK9 gene in implanted human hepatocytes in mice revealed a significant down-regulation of PCSK9 in hepatocytes and the circulating blood [203]. Oligonucleotide-based therapeutics include short interfering RNA (siRNA), that degrade target mRNA, antisense oligonucleotides, that may be working through RNAse-mediated mRNA decay and oligonucleotides-induced alternative splicing.

*siRNA* ***-*** *PCSK9* silencing RNA (siRNA) was formulated in a lipidoid nanoparticle (LNP, Alnylam Pharmaceuticals). Liver-specific siRNA silencing of PCSK9 in mice and rats reduced PCSK9 mRNA levels by 50–70%. The reduction in PCSK9 transcript was associated with up to a 60% reduction in plasma LDL-C levels. In nonhuman primates, a single dose of siRNA targeting PCSK9 resulted in a rapid, durable, and reversible lowering of plasma PCSK9 and LDL-C lasting for 3 weeks after a single intravenous administration [56]. Silencing of the SREBP cleavage activating protein in rhesus monkeys reduces PCSK9 expression by 75% leading to a 50% reduction in LDL-C [80].

The siRNA (ALN-PCS) was tested subsequently in a dose-finding study in 32 healthy participants with an LDL-C above 3 mmol/L, ALN-PCS administered intravenously resulted in dose-dependent reductions in plasma PCSK9 and LDL-C levels, with the highest dose conferring 70 and 40% reductions in PCSK9 and LDL-C levels, respectively, an effect which was sustained for 2–3 weeks after administration. Overall, ALN-PCS was well tolerated with side effects being similar to placebo [54]. Alnylam has recently another phase I clinical trial testing subcutaneously administered ALN-PCS demonstrating a sustained reduction of PCSK9 and LDL-C for up to 180 days after a single injection [55]. The long-term effect is related to stabilization of the siRNA [201].

*Antisense oligonucleotides* (*ASO*) ASOs are short, single-stranded complementary sequences of nucleotides inhibiting protein synthesis by binding to the target mRNA inhibiting protein translation. ASO offers high specificity, but like monoclonal antibodies require intravenous or subcutaneous routes of administration. Two PCSK9-ASOs were initially explored in preclinical trials (for review, see Ref. [172]), but development was stopped after a phase I clinical trial (http://www.clinicaltrials.gov; NCT01082562) because of safety concerns [172].

Nucleic acid analogs that contain at least one monomer in locked conformation (LNA) provide a higher binding affinity and specificity to the target mRNA [88]. LNA ASO reduced the mRNA and protein levels of PCSK9 with a concomitant increase in LDLR protein levels after transfection in HepG2 and HuH7 cells. In mice, tail vein intravenous administration of LNA ASO reduced the level of PCSK9 mRNA by approximately 60%, an effect lasting more than 16 days. Hepatic LDLR protein levels were significantly up-regulated threefold for at least 8 days and approximately twofold for 16 days [71]. In nonhuman primates, LNA ASO targeting PCSK9 produced a sustained reduction of LDL-C after a loading dose and four weekly maintenance doses. PCSK9 mRNA and serum PCSK9 protein were reduced by 85% which resulted in a 50% reduction in circulating LDL-C [111].

Although promising preclinical data are available, the first phase I clinical trial testing the efficacy of SPC5001 (Santaris Pharma)—an ASO with locked RNA nucleotides on both ends of the DNA—was terminated for undisclosed reasons (http://www.clinicaltrials.gov; NCT01350960). One potential explanation for study termination relates to renal side effects, since SPC5001 administered subcutaneously in one volunteer increased creatine levels, white blood cells, granular casts, and caused minimal hematuria on urine microscopy. Kidney biopsy showed multifocal tubular necrosis and signs of oligonucleotide accumulation, all changes being reversible upon termination of SPC5001 administration [198, 199]. Recently, a small molecule compound (R-IMPP) was identified which inhibit translocation of PCSK9 at the level of the 80S ribosome; however, data on the efficiency of such approach for LDL-C management are lacking [143].

*Splice-switching oligonucleotides* (*SSO*) are a new approach to inactivate PCSK9 converting the normal splice form to a natural, less abundant, and inactive, splice variant. Following administration of SSO, an increase of the selected splice form at both the mRNA and protein level was detected when compared to nontreated Huh7 and HepG2 cell lines, with a concomitant increase of the LDL-R protein level. In vivo, full conversion to the splice form was achieved in a reporter system when mice were treated with the specific SSO [156].

Taken together—a number of approaches targeting both intra- and extracellular PCSK9 are under development, some of which passed from the preclinical into clinical testing (siRNA), while others failed (OSA, LNA). Since PCSK9 has multiple intracellular targets [171] one has to see whether long-term results of intra- and extracellular reduction of PCSK9 are advantageous as compared to the extracellular reduction of PCSK9 only.

### Possible pleiotropic effects of targeting PCSK9 and interaction with statins

A recent meta-analysis of >300,000 participants from 49 trials calculated a relative risk reduction for major vascular events per 1 mmol/L (38.7 mg/dL) reduction in LDL-C level of 0.77 for statins and 0.75 for diet, bile acid sequestrants, ileal bypass, and ezetimibe [181]. The authors conclude that “the use of statin and nonstatin therapies that act via up-regulation of LDL-R expression to reduce LDL-C was associated with similar risk reductions of major vascular events per change in LDL-C”. The relative contribution of the cholesterol-independent (or “pleiotropic”) effects of statins for clinical outcomes remains a matter of debate for two main reasons: pleiotropic statin effects are mediated by inhibition of isoprenoids which correlate in humans with LDL-C lowering and are, therefore, hard to quantify. Second, contemporary trials of nonstatin medications, such as the Niemann-Pick C1-like 1 protein inhibitor ezetimibe and the PCSK9 inhibitors, are performed on a statin background. As pointed out in our review, interesting extra-hepatic and/or LDL-C independent effects of PCSK9-inhibition have been described. However, we suspect that—if confirmed in humans—the quantification of their relative clinical importance will face the same challenges as the concept of statin pleiotropy. Some, but not all of these issues, could be addressed by comparing low-dose PCSK9 inhibition with high-potency statin matched in dose for equal LDL-C lowering.

## Studies of PCSK9-inhibition in patients with high cardiovascular risk

The fully human PCSK9-binding antibodies evolocumab and alirocumab have been approved by the FDA (US Food and Drug Administration) and the EMA (European Medicines Agency) in 2015. Both drugs have been tested in high cardiovascular risk patients on top of maximally tolerated statin treatment. Taken together, these studies report an additional reduction of LDL-C (as well as nonHDL and ApoB) by 50–60% and a reduction of lipoprotein Lp(a) by 25–30% [28]. HDL-C and triglycerides are not significantly reduced. The cholesterol content is reduced in the subfractions LDL1, LDL2, and LDL3 + 4 as well as the apolipoproteins CII and CIII and the cholesterol content of very low-density, intermediate-density, and remnant lipoproteins [194]. Studies such as ODYSSEY CHOICE or LAPLACE confirm that the effect of PCSK9 inhibitor is additive to other oral lipid-lowering therapies [184]. The additive effect is consistent with the mechanism of action and the up-regulation of PCSK9 serum concentrations by both statins and fibrates [121].

The available data on safety do not reveal a statistical significant signal [155]. However, further analysis of the ongoing outcome studies is needed to fully assess safety. A potential problem of long-term antibody treatment is the occurrence of autoantibodies. Evolocumab and alirocumab are fully human antibodies and, therefore, theoretically less likely to induce autoantibodies compared to humanized therapeutic antibodies. Only very few cases of anti-drug antibodies have been published to date and no reduction of LDL-C lowering or an off-target effect has been reported, but this topic requires long-term observation [98]. Indeed, the global development program for a third PCSK9 inhibitor, bococizumab, that was tested in large clinical outcome trials, was discontinued because of an unanticipated attenuation of the LDL-C lowering over time, as well as a higher level of immunogenicity and higher rate of injection-site reactions with bococizumab compared to alirocumab and evolocumab [151, 152]. Bococizumab is a humanized antibody that apparently induced more anti-drug antibodies (ADA) compared to the two approved fully human antibodies. Inhibiting ADA can cause a loss of efficacy and very likely contribute to injection-site reactions. Therefore, the topic of ADA will remain important for the future long-term surveillance of the class [98].

Statin-associated muscle symptoms (SAMS) represent a clinical problem that can limit the maximally tolerated dose of a statin [99, 100, 185]. ODYSSEY ALTERNATIVE (alirocumab) and GAUSS-2 (evolocumab) and GAUSS-3 [137, 154] tested PCSK9-antibodies in this complex patient population and found a consistent 50–60% LDL-C lowering response which was superior to ezetimibe.

### Studies of PCSK9-inhibition in patients with familial hypercholesterolemia (FH)

Familial hypercholesterolemia (FH) is a common autosomal inherited condition characterized by a very high risk of atherosclerotic diseases mediated by high LDL-C. FH is under-diagnosed and under-treated [197]. Recent studies have reported the effects of alirocumab and evolocumab in the setting of heterozygous FH (heFH). In RUTHERFORD-2 (Reduction of LDL-C With PCSK9 Inhibition in Heterozygous Familial Hypercholesterolemia Disorder Study-2), treatment with evolocumab against a background of statin/ezetimibe resulted in decreases in LDL-C of 50–60% [57]. LDL-C lowering in heFH was similar irrespective of LDLR mutation status. Data from the ODYSSEY HIGH and the ODYSSEY familial hypercholesterolemia I and II studies with alirocumab showed a similar LDL-C reduction [85]. TESLA (Trial Evaluating PCSK9 Antibody in Subjects With LDL Receptor Abnormalities) [58] and TAUSSIG (Trial Assessing long term USe of PCSK9 Inhibition in Subjects wIth Genetic LDL Disorders) [147] test evolocumab in rare patients with homozygous familial on top of maximally tolerated oral lipid-lowering therapy. As expected from the mechanism of action of PCSK9-inhibition, a range of in LDL-C reduction was observed in LDLR-defective patients and no reduction was seen in LDLR negative patients. Interestingly, Lp(a) was found to be reduced in LDL-R negative patients suggesting mechanisms independent of the hepatic LDLR [183]. The ongoing HAUSER (Trial Assessing Efficacy, Safety and Tolerability of PCSK9 Inhibition in Paediatric Subjects With Genetic LDL Disorders) addressed the important population of patients aged 10–17 years (Clinical Trials Identifier NCT02392559).

To date, lipoprotein apheresis is the only remaining treatment option for individuals with severe FH. The ODYSSEY ESCAPE study (Study of Alirocumabin Patients With Heterozygous Familial Hypercholesterolemia Undergoing Low-density Lipoprotein (LDL) Apheresis Therapy) showed that alirocumab biweekly injection to standard treatment reduced apheresis frequency, the primary efficacy end point, by 75% vs. those who received placebo plus standard therapy [129]. The need for any apheresis treatment at all was completely abolished in 63% of the alirocumab group vs. none of the placebo group.

### Cardiovascular events assessed in safety studies

The ODYSSEY LONGTERM study randomized 2341 patients at high risk for cardiovascular events receiving statins to alirocumab or placebo [100]. At week 24, LDL-C was reduced by 62%. The alirocumab group, as compared with the placebo group, had higher rates of injection-site reactions (5.9 vs. 4.2%), myalgia (5.4 vs. 2.9%), neurocognitive events (1.2 vs. 0.5%), and ophthalmologic events (2.9 vs. 1.9%) [100]. In a post hoc analysis, the rate of major adverse cardiovascular events (death from coronary heart disease, nonfatal myocardial infarction, fatal or nonfatal ischemic stroke, or unstable angina requiring hospitalization) was lower with alirocumab than with placebo (1.7 vs. 3.3%, hazard ratio 0.52, 95% confidence interval 0.31–0.90, nominal *P* = 0.02).

The open-label, randomized OSLER program enrolled 4465 patients who had completed 1 of 12 phase 2 or 3 studies of evolocumab [162]. Evolocumab reduced LDL-C by 61%. After a median observation time of 11.1 months, adverse events occurred with similar frequency compared to placebo, although neurocognitive events were reported more frequently in the evolocumab group. The rate of cardiovascular events at 1 year was reduced from 2.18% in the standard therapy group to 0.95% in the evolocumab group (hazard ratio in the evolocumab group 0.47, 95% confidence interval 0.28–0.78, *P* = 0.003) [162]. This effect was mainly driven by a reduction of coronary revascularizations.

A potential problem of long-term antibody treatment is the occurrence of autoantibodies. Evolocumab and alirocumab are fully human antibodies and, therefore, theoretically less likely to induce autoantibodies compared to humanized therapeutic antibodies. Only very few cases of anti-drug antibodies have been published to date and no reduction of LDL-C lowering or an off-target effect has been reported, but this topic requires long-term observation [98, 193].

An important conclusion of OSLER and ODYSSEY LONGTERM relates to the striking homogeneity of the results of these two programs using different antibodies. Both studies show a very similar and potent LDL-C reduction, are well tolerated, and report a positive signal with regard to cardiovascular events.

A pooled post hoc analysis from ten double-blind alirocumab trials included 4974 patients, of which 104 patients experienced a major adverse cardiovascular event (MACE) [149]. For every 39 mg/dL lower achieved LDL-C, the risk of MACE was 24% diminished. Percent reductions in LDL-C from baseline were inversely correlated with MACE rates [HR 0.71 (0.57–0.89) per additional 50% reduction from baseline] including individuals with very low levels of LDL-C (<50 mg/dL).

The recent GLAGOV study tested the effects of PCSK9 inhibition with evolocumab on progression of coronary atherosclerosis in statin-treated 968 patients with coronary artery disease measured by serial intracoronary ultrasonography (IVUS) imaging [135]. LDL-C was 36.6 mg/dL in the statin + evolocumab group vs. 93.0 mg/dL in the statin group. The PCSK9i evolocumab met its primary end point with a reduction in percent atheroma volume for evolocumab (−0.95%) but not placebo (+0.05%) and a greater percentage of patients demonstrating plaque regression (64.3 vs. 47.3%) after 76 weeks. Post hoc analysis examining the relationship between achieved LDL-C level and change in percent atheroma volume showed a linear reduction down to very low LDL-C levels of 20 mg/dL. No safety signals were observed in the GLAGOV study.

### Endpoint trials with PCSK9 inhibiting antibodies

**Fig. 4:**
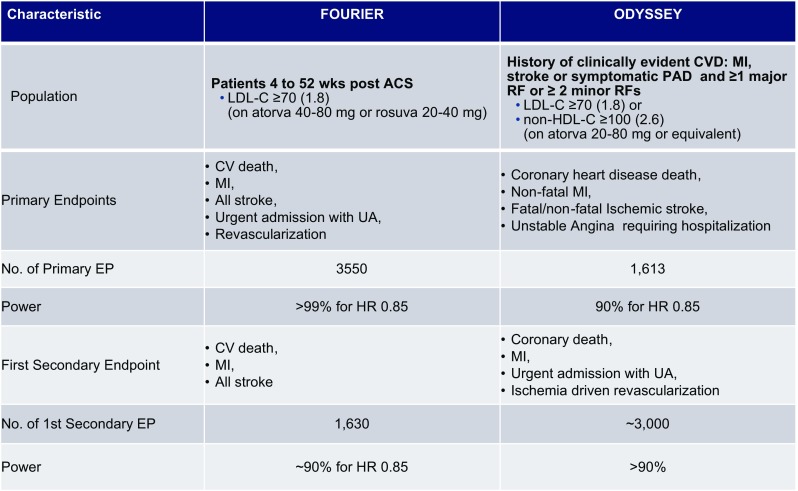
Ongoing large cardiovascular outcome trials in secondary prevention with PCSK9-inhibiting antibodies. The drugs are applied subcutaneously. The recruitment of ODYSSEY outcomes and FOURIER has been completed. The studies are event driven. LDL-C values are depicted in mg/dL (mM/L). *CV* cardiovascular, *MI* myocardial infarction, *UA* unstable angina

The two most important open questions with regard to PCSK9 inhibitor relate to their effects on clinical outcomes and the long-term safety. FOURIER (Further Cardiovascular OUtcomes Research with PCSK9 Inhibition in Subjects with Elevated Risk) is a multinational Phase 3 double-blind, randomized, placebo-controlled trial in 27,564 secondary prevention patients who had either myocardial infarction, an ischemic stroke or symptomatic peripheral artery disease on optimized statin therapy [161] (Fig. 4). Patients were randomized to receive evolocumab s.c. 140 mg every 2 weeks or 420 mg monthly or placebo. LDL-C was lowered from 92 mg/dL (2.4 mM/L) in the statin group to 30 mg/dL (0.78 mM/L) in the patients receiving PCSK9i. Compared to placebo, evolocumab reduced the composite of cardiovascular death, myocardial infarction, or stroke from 7.4 to 5.9% (HR 0.80, 95% CI 0.73–0.88, *P* < 0.001). The results were consistent across key subgroups, including the subgroup of patients in the lowest quartile for baseline LDL cholesterol levels. There was no significant difference between the study groups with regard to adverse events (including new-onset diabetes), with the exception of injection-site reactions, which were more common with evolocumab (2.1 vs. 1.6%). Neurocognitive endpoints were specifically assessed in the EBBINGHAUS (Evaluating PCSK9 Binding antiBody Influence oN coGnitive HeAlth in high cardiovascUlar risk Subjects) involving approximately 1900 patients enrolled in the FOURIER outcomes study. The study showed no differences with regard to executive function (Spatial Working Memory strategy index) and secondary endpoints of working memory, memory function, and psychomotor speed [62].

An important second very large outcome trial testing the PCSK9 inhibitor alirocumab, ODYSSEY OUTCOMES (~18,000 patients recently hospitalized for ACS, NCT01663402), is fully recruited (Fig. 4). The results are expected to be reported in 2018. Together with the FOURIER results, these data will provide a definitive basis for the assessment of the risk and the benefit of this young class of drugs and novel scientific information, e.g., with regard to very low LDL-C serum concentrations.
